# Values Frameworks as Ideal Types: Navigating Ethics Conflicts with Normative Minorities: Values Frameworks as Ideal Types: Response to Critics of “Surrogate Wars"

**DOI:** 10.1007/s10730-025-09559-4

**Published:** 2025-10-15

**Authors:** Autumn Fiester

**Affiliations:** University of Pennsylvania, 423 Guardian Dr., Philadelphia, PA 19102 USA

**Keywords:** Surrogate decision making, Futility, Values-Conflict, End-of-Life, Clinical ethics consultation

## Abstract

In the piece, “Surrogate Wars: The ‘Best Interest Values’ Hierarchy & End-of-Life Conflicts with Surrogate Decision-Makers,” I argue that incommensurable value systems between healthcare providers (HCPs) and surrogate decision-makers (SDMs) lie at the root of many intractable end-of-life treatment disputes. I argue that the most prevalent value system of HCPs might be understood as a “Best Interest Values” (BIV) hierarchy and that this value system is irreconcilable with the set of “Life-Continuation Values” (LCV) held by a sizable minority of families in the United States. I believe that HCPs facing seemingly intractable conflict with SDMs would be aided by understanding their BIV values framework as just one of many cogent values systems in the context of US ethical pluralism. Five preeminent bioethicists provide insightful commentaries on my essay, offering pointed critiques, raising important challenges, and gesturing towards extensions and refinements of my argument. In this essay, I respond to those thoughtful and well-argued commentaries.

In the piece, “Surrogate Wars: The ‘Best Interest Values’ Hierarchy & End-of-Life Conflicts with Surrogate Decision-Makers,” I claim that conflicting value systems between healthcare providers (HCPs) and surrogate decision-makers (SDMs) lie at the root of many intractable end-of-life treatment disputes. I categorize these disparate value systems as Weberian “Ideal Types” to provide a framework for navigating this species of conflict. I argue that the most prevalent value system of HCPs might be understood as a “Best Interest Values” (BIV) hierarchy and that this value system is irreconcilable with the set of “Life-Continuation Values” (LCV) held by a sizable number of families who comprise a “normative minority” in American society. I believe that HCPs facing seemingly intractable conflict with SDMs would be aided by understanding their BIV values framework as just one of other cogent values systems in the context of ethical pluralism in the United States. I worry that there is a pervasive hegemony by the dominant BIV values system against the normative minorities who hold an LCV worldview, and I argue that this hegemony is neither fair nor constructive. The five insightful and well-argued Commentaries offer important challenges, refinements, and extensions of my argument.

The commentary by Janet Malek (2025) offers a pointed criticism of the main theses of my argument, questioning the prevalence and cohesiveness of the BIV and LCV value systems, the usefulness of their application in understanding intractable end-of-life conflicts, and my claims of HCP values hegemony in managing disputes.

Malek’s overarching assessment is that my framing makes “broad generalizations about the values held by clinicians, patients, and surrogates that fail to recognize the diversity of perspectives existing within each of these groups” (2025, p. 464). That depiction makes me wonder whether Malek has fundamentally misunderstood my project, perhaps due to an inadequate articulation of it in my essay. My portrayal of these two contrasting Conceptions of the Good (CoG) is not intended to be an empirical study of two groups of actors in end-of-life conflicts, but instances of what the 20th century German sociologist, Max Weber, calls “Ideal Types” (Weber, 1904). Never one to write plainly, Weber tells us that an Ideal Type is a “synthesis of a great many diffuse, discrete, more or less present…concrete individual phenomena, which are arranged according to …emphasized viewpoints into a unified analytical construct…” (Weber 1904|1949, Shils and Finch 1990, p. 90). An Ideal Type is an “analytical construct” that collates features and elements of some phenomenon in an overly “idealized” way, i.e., it represents that phenomenon in a more integrated or totalizing construct than would occur empirically. An Ideal Type is not meant to mirror reality with empirical accuracy, but to provide a concept that can be used as a reference point, a way of recognizing a phenomenon in its various instantiations. Although Malek acknowledges that “the precise proportion of adherents to each system isn’t central to the paper’s claims or to my rebuttals,” she argues that “[e]mpirical data would be required to accurately determine the prevalence of these respective values systems” (2025, p. 464). But this overly literal read misses the point. My intention in introducing the Ideal Types of the LCV and BIV frameworks is to have these terms enter the “bioethical lexicon” as concepts that articulate values-discordant normative positions that are cogent and rational, but also subjective and perspectival. Naming disparate but cohesive CoGs that can be at play in end-of-life conflicts offers us a scaffolding to understand values discord that rebuts the deleterious appeal to objective morality (a point both Ana Iltis and Takunda Matose emphasized in their Commentaries). The BIV framework does not have privileged access to moral truth but is rather one subjective normative worldview among other possible CoGs, and it stands in stark contrast to an LCV worldview. My hope is that when HCPs and ethics consultants (ECs) understand that their CoG is situated in the context of a diverse tapestry of mores, they will be more circumspect about declaring what is in the “best interest” of the strangers they encounter in end-of-life ethics disputes.

Because my Ideal Types are a tool for understanding normative subjectivity and difference, my project is not undermined by the claim that some patients or families only adhere to parts of the CoG. It would be a gross misuse of this project to treat the LCV and BIV frameworks as a catalogue of any particular individual’s belief system. Ideal Types are not stereotypes, but springboards for dialogue, curiosity, and exploration of another person’s *Weltanschauung*. Devan Stahl illustrates this kind of engagement with these concepts when she focuses her Commentary on the disability community, highlighting tenets of the LCV framework that amplify normative priorities of this group and tenets they are unlikely to hold. Malek need not caution us against “assuming a surrogate believes all these claims simply because they endorse some of them” (2025, p. 465).

While Malek acknowledges that the “BIV system is prevalent among HCP’s and reinforced with the culture of medicine” (2025, p. 464), she seems more skeptical about the existence of normative minorities who hold views antithetical to the BIV system. I’m bewildered by this, not only because I presented data from a variety of sources showing that the LCV cluster captures the various normative perspectives of about 1/3 of Americans, but also because the one claim that most Americans accept is that this country is normatively very polarized. I don’t know whether adherents to the LCV cluster represent 10% or 33% of Americans, but I believe that, whatever the number, normative minorities exist and are at significant risk of having their values and preferences dismissed in disputes with the powerful BIV stakeholders. Normative minorities are not different from other categories of minorities: their minority status creates vulnerability. We take the perspectives of disabled individuals very seriously, although only 13% of the noninstitutionalized population in the US has a disability. In her Commentary, Stahl highlights the relevance of this project for the disability community, reflecting that the contrast between how the BIV and LCV systems understand QoL will immediately resonate with disability bioethicists who fear that discordant QoL criteria can have deadly consequences for disabled patients (Stahl, 2025). That fear is important regardless of how many people experience it.

But Malek is also making a different point: she is arguing that end-of-life conflicts that I attribute to the LCV cluster actually have sources completely unrelated to LCV values. This is why she argues, “The usefulness of these frameworks in resolving conflicts about LST, however, is limited because of the assumption or implication that they can be univocally applied to a certain population of patients and surrogates” (2025, p. 464). Using a series of four case studies that she has drawn from “actual clinical ethics consultations” (2025, p. 465), she depicts SDMs whose resistance to withdrawal of LST appears at first glance to be attributable to a commitment to an LCV CoG, but she believes is not. In two of the cases she describes, the SDMs quickly reconsider their objection to withdrawing LST after dialogue with the EC and move to a comfort care plan of treatment for their loved one, so, indeed, they do not seem to be adherents to an LCV CoG. Those two cases reveal some important insights – which, as a mediator, I wholeheartedly embrace: stakeholders’ starting positions may not reflect their most cherished interests; people can change their minds, especially after considering additional facts; many people have never had the opportunity to reflect on their bioethical values, and a values-elucidation process may give them clarity about views they didn’t know they held; individuals have many values and may decide, upon reflection, to prioritize them differently than they first thought important; our motives may be overdetermined, anchored by more than one reason; and, understanding another party’s reasoning frequently makes us more sympathetic to them (Fiester, 2012; Fiester 2014; Fiester, 2015a, b). All mediators believe that dialogue is indispensable in conflict and can be radically transformative (Bergman, 2015; Morreim, 2018, 2025), and those two cases illustrate this. But Malek seems to believe that I don’t appreciate this. She writes, “As these examples illustrate, the opportunity to understand a surrogate’s underlying reasoning, and therefore to resolve conflicts over continuing LST is lost if such requests are simply respected and accommodated as Fiester proposes” (2025, p. 467). But as a career-long advocate for mediation (Fiester, 2007a, b, 2013; Fiester, 2014b), it would be an anathema to me to be anti-dialogue. What I argue is that “HCPs (and clinical ethicists) have an obligation to engage much more seriously and respectfully with LCV patients and families” (Fiester, 2025, p. 455). I am arguing for *more* dialogue – not less. I agree with Malek that we want to avoid “missed opportunities to find common ground” (Malek, 2025, p. 465).

But I am puzzled by the lesson Malek seems to draw from the other two case studies she presents. In the one case, the SDM elects to continue LST because she believes this best reflects her husband’s values, and in the other, we are told that a chaplain is brought in to think through the SDM’s spiritual beliefs because she is hoping for divine intervention to assist in the recovery of her daughter. In Malek’s original narrative of these two cases, she describes the SDMs as having additional reasons for continuing the LST in their loved ones beyond the tenets of LCV, and she concludes, “Pursuit of these alternative goals and motivations (affirmative and averse) should not be conflated with a true endorsement of LCVs” (Malek, 2025, p. 467). But additional reasons and motivations unrelated to LCV do not negate the existence of an LCV worldview. I sense from Malek a high degree of confidence that her “values elucidation” process produced astute conclusions that the real drivers for these SDMs were unrelated to LCV; but, as a mediator, I urge circumspection. I don’t know if any of these particular SDMs subscribe to LCV, or if any of their loved ones do, but I would caution us about being definitive about other people’s “real” motivations. Mediators see themselves as midwives rather than detectives, and for good reason: our own normative perspectives and biases highly influence our judgment of other people’s motives, it is easy to make false inferences about other people’s reasoning and justifications, and we assume much more about other people than we acknowledge (Fiester, 2015c, 2022a, 2024a; Lechner et al., 2024). To say that “appeals to LCVs are used (consciously or unconsciously) to disguise other goals or motivations that animate the request for continued LST” (Malek, 2025, p. 465) seems deeply problematic to me as a mediator on many levels. Existence of additional non-LCV motivations neither proves the absence of LCV nor reveals motives stronger than the tenets of LCV.

But the lesson I draw from these case studies is how ubiquitous and powerful the BIV framework is in healthcare delivery and ethics consultation. The depictions of the four cases and the corresponding analyses consistently exhibit adherence to the tenets of the BIV CoG, illustrating just what LCV adherents are up against in advocating for the non-BIV values and treatment priorities of themselves and their loved one. The whole framing of the case studies is that continuing LST in any of those instances would be morally wrong and that the HCPs know what is in the best interest of those patients. The certitude of the BIV framework about knowing what is best for patients – in contrast to their SDMs – is striking given what the data show about HCP accuracy. Research shows that hospital-based physicians are the *worst* predictors of patient preferences (Coppola et al. 2001) – much worse than SDMs. And patients *want* SDMs – *not* physicians – to make those decisions, even when they have not selected their surrogate. Of patients who selected a surrogate, 78.2% wanted their surrogate to make treatment decisions whereas 21.8% wanted their physicians to make those choices; for patients who had not chosen a specific surrogate, 66.1% wanted their family to make treatment decisions and 33.9% wanted their physicians (Wendler et al. 2016). Similarly, the Pew Research Center found that ¾ of Americans want their families to make treatment decisions at the end of life (Pew 2009). If “Surrogate Wars” could accomplish just one thing it would be to encourage HCPs and clinical ethicists to operate with a little less certitude about knowing what is best for people they do not know. Malek and I are both in agreement that we must all avoid “making assumptions about others’ values” (2025, p. 469).

Malek’s final line of critique is to challenge my claim that HCPs “hegemonically impose their values on others” (2025, p. 467). Well, there is an entire body of empirical research that demonstrates values imposition by HCPs. In one study published in *Critical Care Medicine*, the official journal of the Society of Critical Care Medicine, physicians *witnessed* other physicians imposing their values. The study authors report, “Physicians had seen colleagues mislead surrogates in a number of ways: Prognosticating with an unreasonable degree of certainty to sway surrogates, intentionally withholding information, and, by one physician’s account, directly lying to surrogates (Brush, Brown, Alexander (Brush, et al., 2012, p. 1084). Some of the HCPs acknowledged their own values imposition, at times expressing remorse: “[S]ome of my greatest regrets as a practitioner have arisen from talking people into things that they were very reluctant to do, and really did not want to do, and only did because I was twisting their arm” (Brush, Brown, Alexander (Brush, et al., 2012, p. 1084). Such values imposition is perceived by SDMs, especially regarding withdrawal of LST. In a separate study in *Critical Care Medicine*, researchers found that 20% of SDMs interviewed one year after the death of their loved one “felt pressure from the staff to hasten their loved one’s death” (Abbott et al., 2001, p. 198), just as the two daughters did in one of Malek’s case studies. HCP values imposition can take different forms. Multiple studies have shown that the options an HCP offers an SDM are highly correlated to the values held by the HCP (Schenker et al. 2012; Needle et al., 2012). Based on their findings, Needle et al. warn, “Physicians should be aware of the potential for personal preferences to influence practice recommendations, and endeavor to elicit and respect family preferences in collaborative decision making” (Needle et al., 2012, p. 2464). The editorial that accompanied this study concluded that “physicians’ beliefs and values are influencing which options they present to surrogate decision-makers, specifically limiting or omitting options that are not in line with the physician’s beliefs” (Kross, 2012, 1586). They added that this problem of values imposition among HCPs requires “clinicians to be aware of their own beliefs and biases when engaging in conversations about end-of-life treatment options with surrogate decision-makers” (Kross, 2012, p. 1586). HCPs *do* “hegemonically impose their values on others” in both overt and subtle ways, and the BIV and LCV frameworks are designed to help HCPs – CEs – avoid values imposition by demonstrating that their normative commitments constitute just one CoG among other legitimate worldviews.

The commentary by Thaddeus Pope very helpfully shows the legal context of the type of disputes I focus on in my article. His review of the current and emerging legal landscape for surrogate decision-making serves as an important resource for ECs, and the utility of his review extends beyond the narrow dialogue we are having about my essay. I have learned a great deal from Pope in this Commentary.

There is one point on which I would like to quibble. Although Pope agrees that “incommensurable value systems (BIV vs. LCV) are a significant cause of [end-of-life] conflicts” (2025, p. 473), he wonders about the portion of conflicts that are truly values-based (as opposed to conflicts generated by other causes). That is a similar line of argument made by Malek, and some of the same arguments I made above apply here. Pope contends that “when surrogates and clinicians disagree, it is usually because the surrogate does not understand the patient’s diagnosis or prognosis. Or the surrogate often misunderstands the role as a surrogate. That is why further intensive communication usually works to resolve these conflicts” (Pope, 2025, p. 473). To anchor this assertion, Pope cites a study by Bakke et al. that conducted focus groups with 40 surrogate decision-makers in San Francisco after their loved one had died (Bakke et al., 2022). But the focus of the Bakke et al. study was not HCP-SDM conflict – it was an exploration of the overall experience of being a surrogate decision-maker and what conditions could have made the role easier. When conflict is described in the study, it is on BIV/LCV grounds. For example, participants reported being unable to defend the values of their loved ones. This problem isn’t caused by SDMs’ confusion about the patient’s clinical condition; it is caused by the power dynamics that subjugate the SDMs’ values. The study reports that SDMs found it “difficult to advocate on behalf of the patient” (Bakke et al., 2022, p. 859). Describing this experience, one SDM said, “I met so many doctors that tend to just tell you [what to do] and then walk out of the room” (Bakke et al., 2022, p. 859). Another SDM expressed frustration at being unable to alter the patient’s HCP-driven treatment course, saying, “Somebody needs to know: ‘No, I don’t think that’s what she wants’” (Bakke et al., 2022, p. 859). In some cases, SDMs felt railroaded by the HCPs. One reported, “I wanted to try my best to keep him alive, but the doctors say ‘Oh, at that age, if you put the tube in you will hurt him even more’” (Bakke et al., 2022, p. 859). Underlying the obstacles these SDMs encountered were value-laden clinical choices made by HCPs. Pope’s cited source does not establish his assertion that “[c]onflict is less often caused by different values” (Pope, 2025, p. 473) The lesson he draws from this study does not correlate with the data the study presents.

While the Bakke et al. study was very small, a recent scoping review corroborated its findings. In a review of over 500 studies involving SDM-HCP conflict in the pediatric context, Parsons and Darlington found that many conflicts were perceived by parents as values-based. They concluded, “Parents identified important themes, in particular their perspective of what constitutes suffering and ’best interest’” (Parsons & Darlington, 2021, p. 981). These are values-based conflicts with the BIV system.

None of this discussion undermines Pope’s contention that my “arguments apply to only a subset of end-of-life healthcare conflicts” (2025, p. 473). That may be true. It is an unanswerable question, given the paucity of data. But what can be established empirically is that conflicts over treatment limitation constitute a large portion of healthcare ethics consultation (HEC). In the study I cited in the main essay, Hauschildt and DeVries found that 63% of HEC involved disputes between SDMs and HCPs and that 2/3 of that 63% involved an HCP who wanted to withhold or withdraw LST and an SDM who wanted to continue LST (Hauschildt and DeVries, 2020). That math ends up being 40% of HEC cases. Other empirical studies found similar results. In one study of HEC over a 15-year period, researchers found that 60% of the 1393 consults involved disputes with SDMs over withholding and withdrawing life support, goals of care, and the patient’s “best interest” (Milliken et al., 2020). A different study found that 70–80% of HEC were disputes with SDM’s over end-of-life care (withholding/withdrawing, DNR, life-sustaining treatment, futility) or goals of care (best interest, quality-of-life, benefits and burdens) (Wasson et al., 2016). So while it is impossible to know what portion of HCP-SDM conflict is values-based, it is clear that the majority of conflicts that come before an ethics consultation service (ECS) are values-based.

Pope provides a thorough and compelling review of the legal backdrop for HCP-SDM conflicts, demonstrating various legal constraints on clinician power, as well as trendlines in the direction of supporting patients and their surrogates. Some of those legal changes are confined to specific jurisdictions (for example, he cites laws only applicable to New Jersey, Texas, and Oklahoma), but some are covered under disability discrimination, which would have national ramifications. The overall picture shows increasing legal support for LCV frameworks articulated by SDMs, and these legal changes will have an impact on SDMs’ ability to advocate for their value system. It will be interesting to see how these changes impact both SDM-HCP futility disputes and the role ECs are allowed to play in those conflicts.

In her thoughtful commentary focusing on disability bioethics, Devan Stahl demonstrates how the LCV and BIV frameworks can be effectively employed to amplify the normative priorities of patients and their SDMs. Her engagement with the LCV construct is a master class in how one maximizes the utility of an Ideal Type, highlighting both points of resonance and departure for a specific subset of patients and families in the quest to make their values visible.

Stahl begins with an exploration of the ways in which certain tenets of the LCV CoG might resonate with people with disabilities, most clearly in the tenet, “Quality-of-life judgment should not be made to determine who lives or dies” (2025, p. 483). In contrast to the LCV understanding of QoL, Stahl argues that the BIV system purports to make an objective assessment of QoL that insults, and can even threaten, disabled lives. Stahl deepens the discussion of QoL through a review of empirical evidence demonstrating that disabled individuals rank their QoL as highly as nondisabled people rank theirs, a fact often disputed (or at least doubted) by non-disabled HCPs. In the disability literature, this discrepancy has been coined the “disability paradox” (2025, p. 481). Stahl shows that the attribution of low QoL and “suffering” to disabled persons by HCPs has a material impact on the kind of treatment they are offered. An early bioethics term, “qualitative futility” (Schneiderman et al., 1990, pp. 952-953), captured an approach to QoL that made the risk of biased judgments against disabled persons palpable. She cites evidence that HCP judgments on QoL relate directly to HCP determinations of futility and HCP-initiated conversations about treatment withdrawal.

While Stahl believes that many individuals with a disability would embrace the QoL tenet in the LCV worldview, Stahl argues many members of the disability community would not subscribe to the tenet, “Human life has intrinsic worth in any state or condition and being alive means having a heartbeat.” Stahl labels this tenet “vitalism” and writes, “It would be a mistake to conflate the concerns of…disability advocates with a preference towards vitalism, or the value of life in any state” (2025, p. 480). She continues, “Vitalism does not well represent many disability advocates’ concerns regarding ableism within healthcare” (2025, p. 480). She argues persuasively that members of the disability community do not necessarily hold that there is no state of life worse than death merely because they believe that many physical and cognitive states do entail valuable existence. In my original argument, I employ the arguments of Terri Schiavo’s parents, the Schindlers, to illustrate tenets of the LCV worldview, but Stahl is right to point out that Terri Schiavo may not have held those same beliefs. While the Schindlers are normative minorities who hold a vitalist perspective, it is possible that they imposed values upon their daughter that might not have been hers. Stahl raises the important concern that overriding an incapacitated patient’s prior values and preferences is an inappropriate form of paternalism that violates the integrity of the patient, and disability advocacy needs to be aware of the risk of attributing LCV views to now-disabled patients. I agree with Stahl that, if Terri Schiavo’s normative views differed from those of her parents and sister, then “allowing her parents’ religious values or preferences for vitalism to supersede her clear prior values and preferences would have been paternalistic and inappropriate” (2025, p. 485). I will only add that one of the contentions of the Schindler family was that they had a relationship with their daughter during those 10 years that she was permanently unconscious. While up until recently, that seemed implausible, new research may vindicate those claims, complicating decisions to remove LST for those in a PVS state on grounds of prior wishes. A recent study in the *New England Journal of Medicine* raises the concern that we underestimate the cognition of some patients in a PVS state (Bodien et al., 2024), supporting claims of family members that their loved ones do engage with the external world. Bodien et al. found that up to 25% of individuals in a coma or persistent vegetative state have an observable response on MRI or EEG when asked to engage in a complex cognitive activity, such as think about a physical task like playing tennis (Bodien et al., 2024). If “cognitive motor dissociation” (i.e., having cognition without the ability to express or act on it) exists among PVS patients, family members may be perceiving real interaction rather than merely projecting it onto their loved ones.

Although generally supportive of empowering SDMs to advocate for loved ones, Stahl urges caution about giving blanket permission to SDMs, and I don’t disagree. Stahl’s main concern involves patient pain. Stahl makes a compelling point that “considerable pain at the end-of-life” is “ethically intolerable” (2025, p. 488), and I agree that demonstrable patient pain is a justification for overriding an SDM’s preference for continued LST. In fact, the recent amendment to the Texas Advanced Directive Act that prohibits using QoL judgments to play a role in LST withdrawal decisions specifically cites pain as a justification for unilateral withdrawal of LST (HB 3162). I completely agree that an SDM does not have the moral authority to block interventions to treat “considerable pain” in an incapacitated patient without a clear directive from the patient themselves. But I also worry that HCPs are very quick to label a patient as being in “considerable pain” and having unacceptable levels of “suffering” when those determinations are more values-based than clinical. This BIV tenet overrides families’ appeals to serve other patient interests even when the amount of discomfort the patient might experience has not been shown to be “considerable.” In a personal example, I was 14 when my mother died of cancer, deeply sedated, and I was denied an opportunity to say “goodbye.” Now as a mother myself, I would certainly choose a few minutes of pain to allow my two children to have the opportunity for closure that a brief (even if painful) interaction would provide. There can be patient-interests greater than comfort, and I believe those should not be dismissed out of hand by BIV-adherent HCPs and ECs.

Stahl’s commentary is a perfect example of how engagement with the LCV and BIV framework can bring into relief important values differences that undergird conflicts about treatment choices, especially at the end of life. She shows that one’s adherence to an LCV or BIV worldview does not have to be totalizing in order for the framework to have utility in values-based discussions.

Whereas Stahl applies the BIV-LCV frameworks to consider the normative perspectives of the disability community, Ana Iltis expands the analysis to explore implications for both “professional ethics” and conscientious objection by HCPs.

Before embarking on a discussion of those two themes, Iltis (2025) offers some preliminary remarks about the frameworks. Importantly, she suggests that, even when individuals hold multiple tenets of a particular CoG, they may believe that a specific tenet must be balanced, or even overridden, by other important interests. For example, an adherent to the LCV system might hold the view that extending life is important but might believe that intractable pain is a good reason to consider treatment-limitation. Those kind of qualifications seem to me to be precisely the way that people manage the various (sometimes competing) commitments of their CoG. Iltis understands the way in which I intend these frameworks to be viewed as Ideal Types rather than a strict litany of the beliefs of a certain population.

Iltis extends the LCV-BIV framework in two philosophically novel and impactful ways. First, she argues that the framework reveals a spurious distinction often touted in the fields of medicine and bioethics between lofty (i.e., objective) “professional values” on the one hand and merely personal (i.e., subjective) ones on the other. Iltis shows us that, at its foundation, the professional *is* personal, and that insight undermines claims that ascribe superior moral authority to “professional ethics.” “Professional ethics” is a way of labeling a set of prescriptive views as objectively correct and values-neutral, which buffers those duties and norms against a charge of subjectivity. But Iltis argues that, under scrutiny, this project fails on two separate grounds.

The first way she challenges the objectivity of “professional ethics” is by showing that the same moral pluralism that exists in the personal realm exists in the professional. There simply isn’t just one CoG among HCPs (or ECs), so the ethics consensus statements of professional societies “obscure difference” (Iltis, 2025, p. 497). Using the example of medical aid in dying (MAID), she demonstrates with empirical evidence that there is no consensus among HCPs, even though the American Medical Association (AMA) states authoritatively that MAID is “fundamentally incompatible with the physician’s role as healers” (AMA 5.7; Iltis, 2025, p. 497). This AMA declaration does not capture the “profession’s” moral views on MAID, but rather reflects the personal moral codes of the group of people who penned it. The hegemonic appeal to “professional ethics” has ignored moral pluralism throughout the history of medicine, as Iltis shows in her striking comparison of professional views of LST withdrawal in the 1980s, where exact opposite argument about withdrawing life-sustaining therapy were fortified under the banner of “professional ethics.” Some ECs are now attempting to reify their subjective HEC bioethical positions by creating HEC “consensus statements” on a whole range of clinical ethics topics, despite clear evidence that no such EC “consensus” exists (Morreim, 2025; Fiester, 2022b; Fiester, 2025).

The second way she challenges the moral authority of “professional ethics” is by showing that ethics codes, policies, and appeals to consensus are not value-neutral but perspectival and subjective. The futility policies that are being created in many US hospitals offer a perfect illustration of this. Those policies do not offer a neutral account of what is ethically appropriate to offer or refuse patients in certain conditions. Those policies reflect the subjective BIV framework. Iltis writes that “futility policies institutionalize and privilege one set of normative commitments and values – the BIV framework – over others” (2025, p. 498). She concludes “So, what is at stake is not a professional objective account of what is right versus a set of personal beliefs. Instead, two sets of personal beliefs compete” (2025, p. 498). I agree with Iltis that futility disputes between HCPs and SDMs “must be described as conflicts between personal beliefs, values, and commitments rather than as conflicts between the professional commitments of healthcare professionals and the personal commitments of patient/surrogates” (2025, p. 499). The ethical subjectivity of futility policies is starting to be recognized, which is why the laws that Pope cites from Texas, Oklahoma, and New York are superseding these subjective policies (Pope, 2025). Pope cites the New York law that states: “[I]f a surrogate directs the provision of life-sustaining treatment…a hospital or individual healthcare provider that does not wish to provide such treatment shall nonetheless comply” (New York, 2010). That is a bald statement that the subjective values of hospitals and providers do not have the moral authority to override the subjective values of patients and SDMs.

Iltis extends the discussion of the LCV-BIV framework in another novel way by using it to complicate the ethical issue of HCP conscientious objection. Given that the values at issue in clinical care are personal, Iltis asks, can BIV physicians recuse themselves through conscientious objection when treating an LCV patient would conflict with their values, or is their recusal better understood as values imposition on those patients and families? At the very least, to invoke “conscientious objection,” Iltis argues, one must be honest about the fact that what is being asked of the HCP conflicts with their personal values, rather than amplifying the HCP values as objective and “hid[ing] behind futility policies that privilege their commitments” (2025, p. 500). In the conscientious objection debate, some argue that personal values must take a backseat to professional values, but that move is blocked when professional values are understood as reflecting subjective normative commitments. I believe that Iltis is correct that there is a need to articulate more precisely the “necessary and sufficient conditions for determining that values imposition has occurred” (2025, p. 500), especially given the gate-keeping power of the BIV framework. Considering the “haves/have nots” of clinical power, it would certainly feel like moral hegemony if BIV HCPs simply refused to provide LST for LCV patients and families on grounds of conscientious objection. My guess is that widespread appeals to conscientious objection for such treatment refusals would invite the law to step in, as it has already in Texas, New York, and Oklahoma. I wonder if appeals to conscientious objection as a way of rejecting LCV choices, if they became widespread, would be short-lived. Regardless, I agree with Iltis’s conclusion: “This is an area where more careful analysis is needed” (2025, p. 500).

In the final commentary, Takunda Matose accepts many of my core claims. He agrees that “end-of-life conflicts are largely the result of clashing value systems” (2025, p. 508) and that the BIV and LCV ideal types offer a lens through which to view these disparate value systems. In fact, he argues that “the reliance on BIV by healthcare providers places them on the same normative terrain as the surrogate decision-makers with whom they are likely to conflict, neutralizing the privilege of expertise” (2025, p. 508). His assessment overlaps with mine, namely, that “the privileging of both healthcare providers and their best interest values system within healthcare creates a continued and unjustified subordination of surrogate decision-makers that threatens to undermine the possibility for meaningful engagement between the two groups” (p. 507). His concern about my argument is that I depict the BIV and LCV worldviews as “incommensurable,” and he argues compellingly that I have been too hasty in this judgment. In fact, he argues that my recommendation for “meaningful engagement” between HCPs and SDMs only makes sense if I am wrong about the incommensurability of the two frameworks. I am persuaded by his argument. Matose also argues that while the “BIV value-system is hegemonic in contemporary Western-style healthcare,” he believes I have been “insufficiently attentive to the ways in which hegemony impedes meaningful engagement with surrogate decision-makers” (p. 514). I believe Matose articulates an important insight here.

Matose defines “incommensurability” as the kind of “deep disagreement” that causes interlocutors to find each other unintelligible. In deep disagreement, there is no possibility of finding common ground because their respective views entail incompatible commitments. Put more philosophically, Matose says that such interlocutors are “unable to resolve conflicts because the kinds of epistemic reasons that each party might offer the other are going to be ineffective” (2025, p. 509). He believes that the clashing values in the BIV and LCV CoGs are not deep disagreements, but merely “serious,” with “enough common ground” to have “meaningful engagement” (p. 507). Matose supports his assessment that the BIV-LCV disagreement is serious but not deep by arguing that “each group thinks it’s important to appeal to many of the same concepts” (2025, p. 511). He argues that “while it’s clear that healthcare providers and surrogate decision-makers often mean different things when invoking concepts like “natural death,” “living,” “killing,” and “futile,” it’s important to note that each group nonetheless appeals to many of the same concepts” (p. 511). We can clearly see that Matose is right about this – at least with some common, disputed terms. Take appeals to “natural” death as an example. When Caterina Gonzalez’s son, Emilio, died on a ventilator, she said he died “naturally, the way God intended” (Cohen, 2007). In contrast, the AMA Code of Ethics states that “when a patient is dying, there may be a choice between returning home to a natural death or remaining in the hospital, attached to machinery (AMA Code of Ethics 5.8, 4). Matose argues, “Both groups appeal to the same concept but disagree on the conceptualization” (2025, p. 511).

While that gives us some hope that the respective worldviews are not philosophically “incommensurable,” there are aspects of end-of-life values systems that seem more like the “deep disagreement” that would qualify as “systematically and persistently incompatible commitments” (2025, p. 509). Take, for example, “futility” or “QoL.” I don’t think it is an accurate description of the conflict to say that LCV adherents are comfortable employing the designation “futile” but simply disagree with BIV adherents about what clinical circumstances constitute the condition of “futile.” I think most LCV adherents would argue that “futility” is beside the point, or worse, is an offense to the dignity of a human being. Using another example, I don’t think the main objection LCV adherents have with using the term “QoL” is that BIV stakeholders underestimate the QoL in their loved ones; LCV adherents believe that this kind of value judgment is inappropriate and misplaced. That makes me worry that adherents to either framework sometimes view the Other as unintelligible, as in: “How could you possibly think like this?” “How could you believe this is what makes life meaningful or worthwhile?” “How could you want this for yourself or anyone else”? This is exactly the tone Fine uses to discuss the SDMs in the case of Mr. J (Fiester, 2025, p. 444). These disagreements seem pretty “deep” to me.

But Matose has an answer to this concern by appealing to what Jennifer Prah Ruger calls “incompletely theorized agreements” (Ruger, 2006). In an incompletely theorized agreement, stakeholders can find some common ground with regard to specific decisions even if those decisions are anchored in competing normative frameworks. Employing this concept, Matose argues that HCPs and SDMs might have “adequate agreement about high level principles” (p. 512), like the principles of “do right by patients,” “respect patient preferences,” or “don’t intentionally cause suffering,” to make dialogue possible between stakeholders. As a mediator, this sounds right to me. In fact, mediators refer to the idea “interests” to talk about the kind of universal needs, concerns, values or stakes that underlie disagreements and can reveal common ground, or at least shared humanity, among disputing parties (Fiester, 2015a, b). All mediators see hope for gaining traction in a dispute through the vehicle of identifying the parties’ interests. In that way, our positions align about the possibility of meaningful engagement.

Finally, I agree with Matose that competing value systems between HCPs and SDMs are only one source of “surrogate wars.” He rightly identifies a “recalcitrant prioritization of the decisions of healthcare providers,” which he views as being “as big a problem as the content of their underlying belief system” (2025, p. 514). He argues that “healthcare providers are privileged as decision-makers at the expense of surrogate decision-makers” (p. 514). I believe Matose rightly perceives the “rules of engagement” for HCPs. Admittedly, HCPs are fully committed to honoring the choices of conscious competent patients – regardless of how antithetical those choices are to what an HCP believes is best. That respect for patient autonomy is part of the fabric of clinical life. The clash of worldviews between BIV and LCV only matters because of the involvement of SDMs. That dispute is about which party – the HCP or the SDM – is doing right by the patient in the absence of a conscious competent patient-decision-maker. HCPs have no trouble accepting patient choices born out of radically different CoGs – which is why they fully accept the refusal of lifesaving blood products among members of the Jehovah’s Witness. But HCPs operate with a pervasive skepticism (and maybe condescension) regarding SDMs with incommensurable worldviews. As Matose puts it: “The result is that surrogate decision-makers are subordinated while their value system is dismissed” (2025, p. 514).

What is peculiar about this is that the data demonstrate that patients *want* their SDMs in the decision-making role. Patients are well aware of the CoGs of their loved ones. Who is confused about where the values-based disagreements will occur around the Thanksgiving table? Meaningful engagement with the SDMs will mean granting the benefit of the doubt that the values articulated by the SDMs mirror the values of the patient, in the absence of a preponderance of evidence to the contrary. Afterall, if a patient has serious reservations about the prospect of some family member or friend being assigned the role of the SDM (after a lifetime of Thanksgivings as data points), the patient can take easy steps to prevent their involvement in their healthcare: they can name a healthcare proxy, they can write an advance directive, they can document that certain individuals are *not* to be granted SDM status.

In summary, I agree with Matose that conflicting value systems do not preclude meaningful engagement between HCPs and patients, and they need not preclude meaningful engagement between HCPs and SDMs. “Surrogate wars” occur because HCPs feel entitled to engage in values hegemony with SDMs that they would not dare with conscious competent patients. HCPs delegitimize the CoGs of SDMs because they view themselves as best positioned to know what is in the “best interest” of the incapacitated patient. My hope is that by engaging with LCV SDMs – trying to understand the LCV worldview, using curiosity and open-mindedness to explore why the SDM believes the LCV framework best captures the patient’s values – the HCP can put their own BIV framework in the context of one CoG among others, and quite possibly (maybe even likely) not the CoG of the patient they are treating. Entering the BIV/LCV framework into the bioethical lexicon provides HCPs (and ECs) a mechanism to critically reflect on the moral certitude they bring to end-of-life futility disputes.
